# Editorial: Computational Methods for the Description of Intermolecular Interactions and Molecular Motion in Confining Environments

**DOI:** 10.3389/fchem.2022.941269

**Published:** 2022-05-31

**Authors:** Heribert Reis, Piotr Żuchowski, Sonja Grubisic

**Affiliations:** ^1^ Institute of Chemical Biology, National Hellenic Research Foundation, Athens, Greece; ^2^ Institute of Physics, Nicolaus Copernicus University in Toruń, Toruń, Poland; ^3^ Institute of Chemistry, Technology, and Metallurgy, National Institute of Republic of Serbia, University of Belgrade, Belgrade, Serbia

**Keywords:** molecular confinement, nuclear motion, intermolecular interactions, molecular simulation methods, carbon nanotubes, SAPT

Methods of computational chemistry have become increasingly important in the last decades for the investigation of interaction and dynamics of small molecules enclosed in larger, micro- and mesoscopic structures, as witnessed by a large number of publications in a large number of diverse fields, such as design of pharmaceutical drugs Roy (2019), investigations on the mechanisms of drug delivery Garifalo et al. (2020), design of novel materials for bioimaging Bonačić-Koutecký and Antoine (2019); Combes et al. (2021), catalysis Li et al. (2021), gas storage Kundu et al. (2016), information and communication technologies Ghosh et al. (2021), but also on fundamental research, for example in astrophysics Puzzarini and Barone (2020); de Lara-Castells and Hauser (2020). Molecular confinement in single molecular structures may lead to new and unexpected phenomena, which are not easily predicted by classical theories of confinement in bulk materials. Due to the small to medium size of these confining structures, they can be treated in principle by modern computational methods with moderate to high accuracy, thus allaying the need for difficult experiments. However, new techniques and methodologies may be required for successful treatment of interactions and dynamics of molecules in confining structures. It is the purpose of this Research Topic of ten original research contributions to highlight current directions and present some applications of computational methods for molecular systems in confining environments.

Recent developments and an outlook on possible developments in the future on computational approaches for the treatment of small clusters comprised of He atoms or H_2_ molecules in carbon nanotubes are reviewed in (de Lara-Castells and Mitrushchenkov). For an accurate description of these dispersion–dominated systems, difficult to treat by standard quantum-mechanical methods, a multi-scale approach using a DFT-based symmetry-adapted perturbation theory (SAPT) method is described. A second, wave-function based method, also allows the computation of shallow bound states in these systems.

Two contributions attest to the importance of research on the behaviour of small molecules in the confining environment of natural proteins in pharmaceutical and medicinal research. Zhang et al. use classical simulation techniques, i.e. molecular docking, molecular dynamics simulations and adaptive steered molecular dynamics simulations to investigate the interactions of two inhibitors drugs with the N-terminal Human Maltase Glucoamylase, in an effort to advance the search for effective drugs to treat diabetes 2.

Jiang et al. combine experiment and computational molecular simulations to study the interactions of two alternaria mycotoxins with a single-chain antibody fragment.

Another study using classical molecular dynamics simulations to investigate a Research Topic of confinement of biological importance is presented by Vasiliu et al. They study the interactions between positively charged polyamines which act as counterions to negatively charged DNA fragments in cells using microsecond molecular dynamics simulations.

A contribution from the field of chemical engineering on the effect of confinement on solubilties of gases in ionic liquids enclosed by nanopores is reported by Sun et al., who improved the efficiency and applicability of electrolyte perturbed-chain statistical associating fluid theory–density functional theory (ePC-SAFT-DFT). The newly devised method was then applied to study the effect of several variables on the solubiltity of CO_2_ in ionic liquids confined in silica nanopores, which led to new conclusions how to increase the solubility of CO_2_ in ionic liquids.

Three studies are concerned with the effect and analysis of intermolecular interactions, clearly a dominant Research Topic for confined systems. These interactions may lead, for example, to hydrogen bonding, which are the focus of the first two papers, or other types of noncovalent bondings, such as halogen or chalcogen bonds.

In the first paper, Kaczmarek-Kedziera et al. present a study on the effect of intermolecular interactions, specifically hydrogen bonding, on two optical properties, namely electronic one- and two-photon absorption, applying quantum-mechanical methods on hydrogen bonded intermolecular complexes of two 4-substituted N,N′-diphenyl-urea or N,N′-diphenyl-thiourea with one central squaraine molecule. Using different substituents in position 4 of phenyl the acidity of the N-H protons can be varied.

In the second paper, the effect of a confining environment on the IR spectra of two hydrogen-bonded intermolecular complexes, namely HCN—HCN and HCN—HNC, is studied by Chołuj et al. The spatially confining environment is simulated by embedding the complexes in an external cylindrical harmonic oscillator potential, and the harmonic frequencies are computed with high-level *ab-initio* methods The simulated confinement leads to substantial changes of both transition intensities and vibrational frequencies.

In the third study Szczesniak and Chalasinski, report an interesting analysis of a number of complexes with a more general type of intermolecular interactions, i.e., weak electron-donor electron-acceptor complexes including examples of chalcogen and halogen bonds. With the help of Symmetry Adapted Perturbation Theory (SAPT), they challenge the prevailing interpretation of these interactions as of an electrostatic nature. Instead, they argue that exchange or Pauli repulsion is more relevant.

A question relevant for astrochemistry is studied by Upadhyay and Meuwly, namely the distribution of energy released due to exothermic reactions on amorphous solid water. Specifically, they investigate the energy transfer during energy relaxation of the product in the recombination reaction CO(^1^Σ^+^) + O (^1^D) → CO_2_(^1^ $\Sigma_{g}^{+}$ ) within and on the surface of amorphous solid water and the surrounding water molecules.

Finally, a novel embedding simulation method, called real-time time-dependent block orthogonalized Manby-Miller embedding approach is implemented by de Santis et al., and applied to study the X-ray absorption spectra of model systems comprised of fluoride and chloride ions confined by 8 water molecules ([X@(H_2_O)_8_]^−^, X=Cl,F).

The field of computational techniques to treat confined systems in the extended definition used here is very large and extremely varied, and this Selection can only present a limited number of recent applications and developments in this Research Topic. Nevertheless, we believe that the selected papers are evidence of the vibrant activity of current research in this field. We expect that the role of computational treatments of confined systems will become more important in the future, due to the increasing search for systems with specific properties for applications on the microscopic and nanocscopic scale, as well as due to the steadily increase of computing power and the ongoing development of sophisticated modelling techniques.

## References

[B1] Bonačić-Koutecký V. Antoine R. (2019). Enhanced Two-Photon Absorption of Ligated Silver and Gold Nanoclusters: Theoretical and Experimental Assessments. Nanoscale 11, 12436–12448. 10.1039/C9NR01826C 31162509

[B2] Combes G. F. Vučković A.-M. Perić Bakulić M. Antoine R. Bonačić-Koutecky V. Trajković K. (2021). Nanotechnology in Tumor Biomarker Detection: The Potential of Liganded Nanoclusters as Nonlinear Optical Contrast Agents for Molecular Diagnostics of Cancer. Cancers 13, 4206. 10.3390/cancers13164206 34439360 PMC8393257

[B3] de Lara-Castells M. P. Hauser A. W. (2020). New Tools for the Astrochemist: Multi-Scale Computational Modelling and Helium Droplet-Based Spectroscopy. Phys. Life Rev. 32, 95–98. 10.1016/j.plrev.2019.08.001 31443981

[B4] Garofalo M. Grazioso G. Cavalli A. Sgrignani J. (2020). How Computational Chemistry and Drug Delivery Techniques Can Support the Development of New Anticancer Drugs. Molecules 25, 1756. 10.3390/molecules25071756 PMC7180704 32290224

[B5] Ghosh D. Ivanov S. A. Tretiak S. (2021). Structural Dynamics and Electronic Properties of Semiconductor Quantum Dots: Computational Insights. Chem. Mat. 33, 7848–7857. 10.1021/acs.chemmater.1c02514

[B6] Kundu A. Piccini G. Sillar K. Sauer J. (2016). Ab Initio Prediction of Adsorption Isotherms for Small Molecules in Metal-Organic Frameworks. J. Am. Chem. Soc. 138, 14047–14056. 10.1021/jacs.6b08646 27748594

[B7] Li S. Wang Y. Qin B. Zhou Z. Zhou S. Li K. (2021). “Mechanistic Studies toward the Rational Design of Oxide Catalysts for Carbon Dioxide Hydrogenation,” in Annual Reports in Computational Chemistry. Editors Dixon D. A. Chair R. R. (Amsterdam: Elsevier), Vol. 17, 211–270. 10.1016/bs.arcc.2021.09.001

[B8] Puzzarini C. Barone V. (2020). A Never-Ending Story in the Sky: The Secrets of Chemical Evolution. Phys. Life Rev. 32, 59–94. 10.1016/j.plrev.2019.07.001 31320317

[B9] K. Roy (Editor) (2019). In Silico Drug Design. Repurposing Techniques and Methodologies (Cambridge, Massachusetts, USA: Acadenic Press).

